# Relationship between the dissemination of small ruminant lentivirus infection in goat herds and opinion of farmers on the occurrence of arthritis

**DOI:** 10.1371/journal.pone.0204134

**Published:** 2018-09-13

**Authors:** Michał Czopowicz, Olga Szaluś-Jordanow, Marcin Mickiewicz, Agata Moroz, Lucjan Witkowski, Andrzej Bereznowski, Iwona Markowska-Daniel, Emilia Bagnicka, Jarosław Kaba

**Affiliations:** 1 Laboratory of Veterinary Epidemiology and Economics, Faculty of Veterinary Medicine, Warsaw University of Life Sciences, Warsaw, Poland; 2 Department of Small Animal Diseases with Clinic, Faculty of Veterinary Medicine, Warsaw University of Life Sciences, Warsaw, Poland; 3 Institute of Genetics and Animal Breeding, Polish Academy of Sciences, Jastrzębiec, Magdalenka, Poland; Istituto Zooprofilattico Sperimentale delle Venezie, ITALY

## Abstract

Small ruminant lentivirus (SRLV) infection manifests itself mainly with chronic progressive arthritis affecting mainly carpal joints. The data from serological and questionnaire surveys were retrospectively analyzed to determine how the dissemination of SRLV infection in the herd influenced farmer’s subjective opinion on the occurrence of swelling of carpal joints (considered as a proxy of arthritis). Between 1996 and 2017 153 different Polish dairy goat herds counting at least 20 adult goats were serologically screened for CAE and their owners were asked about their opinion on the occurrence of arthritis (never, rarely, often). Of them 73 SRLV-seropositive herds, in which true seroprevalence had been estimated, were included in the analysis. The ordinal logistic regression model was developed to determine the relationship between the true within-herd seroprevalence and the probability that the farmer would observe arthritis in the herd never, rarely or often. True within-herd seroprevalence ranged from 0.2% to 100% with the median of 34.6%. Farmers declared not to have observed arthritis in 40 (54.8%) herds, to have seen it rarely in 9 (12.3%) of herds, and to have observed it often in 24 (32.9%) of herds. The model proved that the probability of observing goats with carpal arthritis in the herd was significantly linked to the true within-herd seroprevalence (OR = 1.058, CI 95% from 1.037 to 1.078; p<0.001), but this relationship was not linear and SRLV infection proved to remain unapparent to farmers even when a considerable part of the herd had already become infected. Concluding, the study shows that when the farmer realizes that goats in the herd suffer from arthritis, SRLV infection is almost certainly already widespread in the herd.

## Introduction

Caprine arthritis-encephalitis (CAE), caused by a small ruminant lentivirus (SRLV) infection, is a widespread transmissible disease of goats with a considerable negative impact on dairy production [1,2,3]. The disease emerged in Poland in early nineties of the 20^th^ century and has become widespread in Polish goat population over the next decade from roughly 30% in 1996 to 70% in 2007 [4]. Recent studies have revealed that goats in Poland are infected with SRLV subtypes A1, A12, A13, B1 and B2 [5] as well as with two novel subtypes A16 and A17 [6], and one goat may be co-infected with viruses belonging to group A and B [5,6]. Progressive arthritis, mainly involving carpal joints, is the most prominent clinical sign of CAE [7]. Nonetheless, as it develops slowly and only in a part of infected goats [7,8], SRLV infection disseminates in the herd long before first symptomatic goats are noticed. Serological screening of the herd is therefore the only method of early detection of the disease [9]. However, farmers are reluctant to spend money on laboratory screening of apparently healthy herds, since they believe they are sufficiently experienced and observant to capture the disease in its early stage. Even though, the whole knowledge of CAE pathophysiology unambiguously indicates that they are wrong, the straightforward epidemiological evidence is lacking. Therefore, we retrospectively analyzed data from serological and questionnaire surveys to determine to what extent farmer’s subjective opinion on the occurrence of arthritis in their goats corresponded with the true prevalence of SRLV infection in the herd.

## Materials and methods

The study was based on records of Polish dairy goat herds which our team had visited in last 20 years (1996–2017) within the frame of the routine voluntary CAE diagnostic program. The study was approved by the 3^rd^ Local Ethical Committee in Warsaw (Approvals No. 44/2009, 31/2013). In each herd informed consent for participation was granted by the farmer. The herds were scattered over the entire territory of Poland, with the highest concentration in western part of the country. To be enrolled in the analysis a herd had to count at least 20 adult goats (i.e. older than 1 year) and must not have been screened for SRLV infection before. If a herd was visited more than once in this time period the record from the first visit was included in the analysis. In each herd a standardized interview was conducted with its owner (thenceforth referred to as the farmer) always by the same board-certified specialist in small ruminant health management (JK). The answers to following questions were included as variables in the further analysis:

1. How often adult goats with swollen carpal joints are observed in the herd?–included as the ordinal response variable named “the farmer’s opinion on the occurrence of arthritis in the herd”. Possible answers were: never, rarely (if the farmer saw single goats with swollen joints in the herd but did not consider it as a serious problem for herd health), and often (if the farmer considered goats with swollen joints as a considerable burden for herd health).
2. How many adult goats (males and females) are currently kept in the herd?–included as the additional explanatory variable called “herd size”. This numerical variable was included to control for the known positive relationship between herd size and the occurrence of SRLV infection [4,10] as well as to adjust the model by possible subjectivity of carpal arthritis perception in herds of various size.
3. How many years ago the herd was established and for how long has it remained under the farmer’s direct supervision?–included as the additional explanatory variable called “farmer’s experience in goat management”. First, this numerical variable was included to control for the gradually increasing seroprevalence when infected goats are kept in the herd [11]. Secondly, we wanted to take into account farmer’s growing experience in goat management as more experienced farmers are more likely to identify arthritic goats quicker and regard them as a serious problem for herd health.

Then, adult goats in the herd were blood-sampled–either all adult goats in the herd or the representative sample (for sample size calculation see below) selected randomly from the list of ear-tag numbers or goat names provided by the farmer (precisely, small pieces of paper with ordinal numbers of goats were drawn manually by one of investigators from a box and the selected goats were identified by the farmer). Blood was collected from the jugular vein to dry 10ml tubes, kept overnight at room or fridge (2–8°C) temperature to allow for clotting, and centrifuged at 3000 ×g for 10min. The harvested serum was stored in 2ml vials at -20°C until testing.

Serum samples were screened with one of two whole-virus immunoenzymatic assays which were available on the marked in a given time period: Checkit CAEV/MVV (Dr. Bommeli AG, Bern, Switzerland) before 2007 and then ID Screen MVV-CAEV Indirect Screening test (ID.vet Innovative Diagnostics, France). Both ELISAs were performed according to manufacturers’ manuals. Sensitivity (Se) and specificity (Sp) was 98.6% and 99.3% respectively for Checkit ELISA [12], and 91.7% and 98.9%, respectively for ID Screen ELISA [13]. Apparent within-herd seroprevalence (AP) was the proportion of seropositive adult goats in the herd. True within-herd seroprevalence (TP) was calculated according to the formula:

$$TP=\frac{AP+Sp-1}{Se+Sp-1}$$ (1)

TP was calculated only if the goat sample selected in the herd was equal or bigger than the required sample size (n) sufficient to estimate within-herd seroprevalence assuming expected true within-herd seroprevalence of 50% (eTP), precision of the estimation (L) of 10%, level of confidence of 95% and the aforementioned Se and Sp of the ELISA used. This sample size was calculated with adjustment for a herd size (N) using the following formula [14,15]:

$$n=\frac{N\times {\left(\frac{1.96}{L}\right)}^{2}\times \frac{\left[Se\times eTP+(1-Sp)\times (1-eTP)]\times [\left(1-Se\times eTP)-(1-Sp)\times (1-eTP\right)\right]}{{\left(Se+Sp-1\right)}^{2}}}{N+{\left(\frac{1.96}{L}\right)}^{2}\times \frac{\left[Se\times eTP+(1-Sp)\times (1-eTP)]\times [\left(1-Se\times eTP)-(1-Sp)\times (1-eTP\right)\right]}{{\left(Se+Sp-1\right)}^{2}}}$$ (2)

TP was the main explanatory variable included in the analysis.

Numerical variables (i.e. herd size and farmer’s experience in goat management) were inspected for normality of distribution using the Shapiro-Wilk W test. As this assumption was constantly violated they were presented as the median, interquartile range (IQR) and range, and compared with each other using the Spearman rank-ordered correlation coefficient (r_s_), and between groups using the Kruskal-Wallis H test and a Conover–Iman post-hoc test [16]. Ninety five per cent confidence interval (CI 95%) for proportions was calculated according to the Wilson score method [17] and for r_s_ according to Bonnett and Wright [18].

To determine the relationship between the true within-herd seroprevalence (TP) and the probability that the farmer would observe arthritis never (P = 0), rarely (P = 1) or often (P = 2) the following ordinal logistic regression model was built [19]:

$$Y(P=2)=\frac{1}{1+e^{\left(a_{2}-b_{TP}\times TP-b_{2}\times X_{2}-b_{3}\times X_{3}\right)}}$$ (3)

$$Y(P=1)=\left(\frac{1}{1+e^{\left(a_{1}-b_{TP}\times TP-b_{2}\times X_{2}-b_{3}\times X_{3}\right)}}\right)-Y(P=2)$$ (4)

Y(P=0)=1−Y(P=1)−Y(P=2) (5)

where e was Euler's number equal to ≈2.718; a_1_ and a_2_ were the intercepts for level 1 (arthritis rarely observed) and 2 (arthritis often observed), respectively; b_TP_ was ordinal regression coefficient of the main explanatory variable TP. b_2_ and b_3_ were ordinal regression coefficients of the additional explanatory variables “herd size” and “farmer’s experience in goat management”, respectively, added to the model in the course of a stepwise procedure. For intercepts and regression coefficients standard errors (SE) and CI 95% were reported. The final model was build according to manual forward stepwise procedure: first the model was run only with TP. Then, “herd size” and “goat management experience” were entered into the model, separately and together, and b_TP_ was inspected–the additional explanatory variable was retained if it changed the relationship between TP and arthritis by at least 10%. Goodness-of-fit was evaluated using Pearson statistic, deviation measure and Nagelkerke’s pseudo-R^2^. The assumption that the regression coefficients (so the odds) were equal in all three categories of arthritis was evaluated using the test of parallelism. On the basis of b_TP_ odds ratio (OR) for TP with CI 95% was computed as:

$$OR=e^{b_{TP}}$$ (6)

$$CI\;95\%=e^{b_{TP}\pm 1.96\times SE}$$ (7)

A two-tailed significance level (α) of 0.05 was assumed in all statistical tests. The analysis was carried out in the Microsoft Office Excel 2013, Statistica 12 (StatSoft Inc., Tulsa, OK) and IBM SPSS Statistics, and figures were prepared in the two former packages.

## Results

Between 1996 and 2017 we visited and tested 273 different goat herds in total and 153 of them (56.0%) counted at least 20 adult goats. Of those 153 herds in 104 herds at least one goat tested seropositive for SRLV, which yielded an apparent herd-level seroprevalence among herds counting at least 20 adult goats of 68.0% (CI 95% from 60.2% to 74.8%). In 31 of 104 seropositive herds a sample collected was too small to estimate TP (we could only qualitatively conclude that they were infected) so 73 herds (47.7% of 153 herds) were eventually included in the analysis. In 46 herds (63.0% of 73 herds) all or virtually all (>90%) goats were blood-sampled. In the remaining 27 herds (37.0%) a representative sample was tested (S1 Table). In 11 of 73 herds (15.1%) enrolled in the analysis goats were kept in close contact with sheep.

Herd size ranged from 20 to 1150 goats with the median of 62 (IQR from 38 to 98) goats. True within-herd seroprevalence ranged from 0.2% to 100% with the median of 34.6% (IQR from 8.3% to 81.0%). The time which had elapsed since the herd was established ranged from 1 to 35 years with the median of 9 (IQR from 4 to 14) years and in all cases the herd had been run by the same farmer since it was established so this variable was a good proxy of the farmer’s experience in goat management. Farmers declared not to have observed arthritis in 40 (54.8%) herds, to have seen it rarely in 9 (12.3%) of herds, and to have observed it often in 24 (32.9%) of herds (S1 Table).

**Fig 1:**
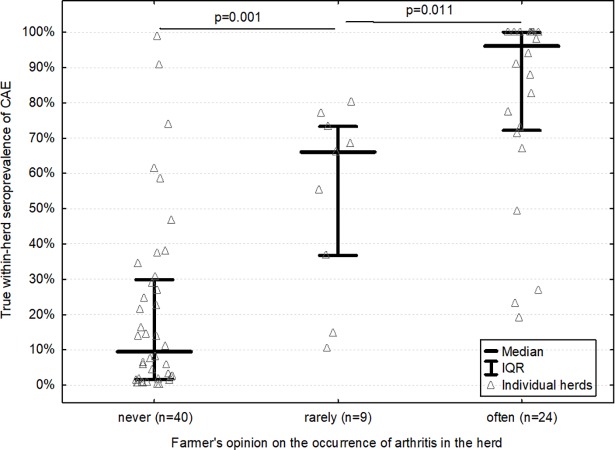
True within-herd seroprevalence of SRLV infection in goat herds classed according to the farmers’ opinion on the occurrence of arthritis (described as goats with apparently swollen joints) in the herd.

TP proved to differ significantly depending on the farmer’s opinion on the occurrence of arthritis (p<0.001) (Fig 1). TP was also linked to the herd size (r_s_ = 0.33, CI 95% from 0.10 to 0.53; p = 0.004) and the farmer’s experience in goat management (r_s_ = 0.25, CI 95% from 0.02 to 0.46; p = 0.030). In turn, these two additional explanatory variables turned out to be also significantly related to the farmer’s opinion on the occurrence of arthritis in the herd (p<0.001 and p = 0.017, respectively). Therefore, they satisfied statistical requirements for potential confounders and they were entered into the ordinal logistic regression model.

Ordinal logistic regression model proved that the probability of observing goats with arthritis in the herd was significantly linked to TP (OR = 1.058, CI 95% from 1.037 to 1.078; p<0.001), but was not related either to the herd size or the farmer’s experience in goat management, which remained insignificant (p = 0.140, and p = 0.278, respectively) and changed the regression coefficient of TP by only 7% and 5%, respectively. Therefore, the two additional explanatory variables were dropped from the final ordinal logistic regression model. The model fit the data well: Pearson chi-square tests and deviation measures were insignificant (p = 0.193 and p = 0.990, respectively) and Nagelkerke’s pseudo-R^2^ was 0.581. The assumption of parallelism was also satisfied (p = 0.885). Detailed results of the ordinal logistic regression model are given in Table 1 and the interim models containing additional explanatory variables in S2 Table.

**Table 1:** Ordinal logistic regression model estimating the probability of observing arthritis in the herd at particular true within-herd seroprevalence level.

Variable	Regression coefficient (b)	Standard error (SE)	95% confidence interval	Wald χ^2^ statistics	p-value
Arthritis					
never observed (0)	-	-	-	-	-
rarely observed (1)	2.789	0.592	1.629, 3.949	22.22	<0.001
often observed (2)	3.850	0.703	2.472, 5.228	29.98	<0.001
True within-herd seroprevalence (TP)	0.056	0.010	0.036, 0.075	31.30	<0.001

**Fig 2:**
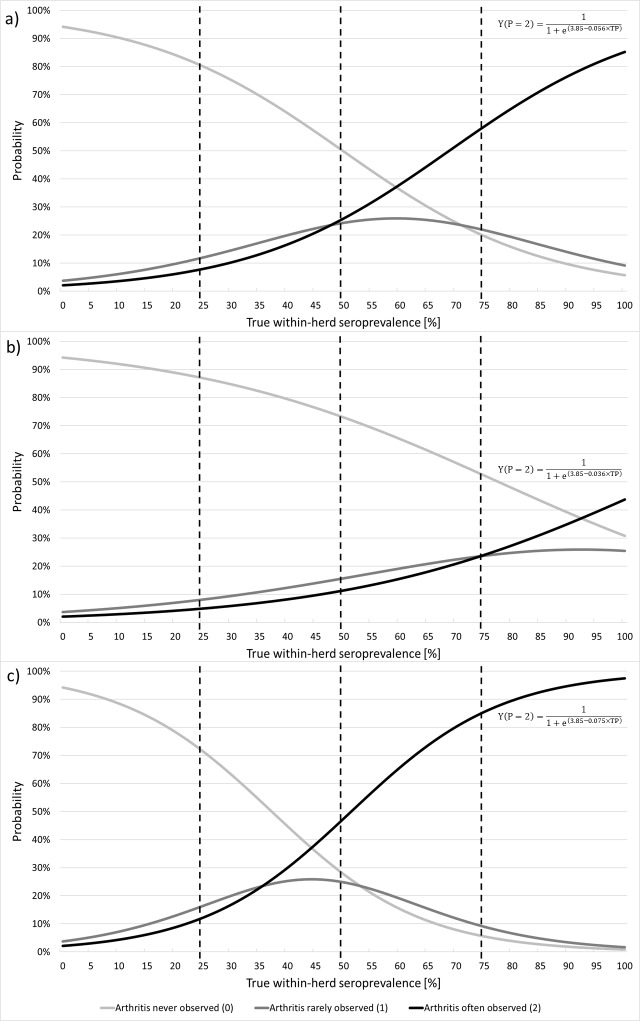
Probability of observing arthritis in the herd never (light grey line), rarely (dark grey line) or often (black line) estimated by the ordinal logistic regression model based on the true within-herd seroprevalence of SRLV infection in goat herds: a) model with regression coefficient of the true within-herd seroprevalence of 0.056, b) model with regression coefficient of the true within-herd seroprevalence of 0.036 (the lower 95% confidence limit of the estimation), c) model with regression coefficient of the true within-herd seroprevalence of 0.075 (the upper 95% confidence limit of the estimation).

On the other hand, the relationship between probability of observing goats with arthritis in the herd and TP was not linear and the main clinical sign of SRLV infection proved to remain generally unapparent to the farmer even when a considerable part of the herd had already been infected (Fig 2): when already one fourth of goats were infected the farmer was 80% (CI 95% from 71% to 87%) likely not to see arthritis at all; this probability was still as high as 50% (CI 95% from 28% to 73%) when a half of goats were infected, and about 20% (CI 95% from 6% to 52%) when three fourths of goats were infected. In turn, when the infection had already affected a half of goats in the herd the farmer was still only 26% (CI 95% from 11% to 48%) likely to consider arthritis as a serious problem for the herd health.

## Discussion

In our study we wanted to show the relationship between the dissemination of SRLV infection in a herd and probability that a farmer would notice that something wrong is going on. For this purpose we used seroprevalence of SRLV infection as a proxy of SRLV infection and carpal joint swelling as a proxy of clinical signs of SRLV infection. We did not aim to determine how accurate is farmer’s observation in diagnosing SRLV infection. Therefore, only SRLV-affected herds were included in the analysis and within-herd seroprevalence of SRLV was used as an explanatory variable.

Our study clearly shows that the development of the most prominent clinical sign of SRLV infection, or more precisely, the moment when a farmer begins to see it, is considerably delayed compared with the dissemination of SRLV infection in the herd. No matter how experienced the farmers were and how small or large herd they owned, they were unable to capture the disease early–the lowest prevalence at which farmers saw some goats with arthritis was 10% (Fig 1) and the estimated probability of noticing arthritis remained around only 25% at best up to the moment when a quarter of goats were already seropositive (Fig 2). Obviously, the likelihood that farmers notice a goat with swollen joints must, to some extent, depend on their experience, observant skills and time they spend with their animals. Farmers included in our study were fairly experienced–most of them (66%, S1 Table) had owned goats for more than 5 years. Moreover, except for a few largest herds, farmers were directly responsible for taking care of goats, which was their full-time job–they milked them, led them to and from the pasture, cleaned the barn, assisted in parturitions–so every day they used to see all their goats and had many occasions to spot any alarming signs. Most of farmers in our study had set up their herds as hobbyists of goat farming and it was clear for us that they had personal, emotional attitude to their goats, often expressing itself by knowing them all by name (even in herds counting nearly 100 goats!). It has been well evidenced that farmers’ attitude towards animals has strong influence on their performance and welfare [20,21] and we had no doubts that farmers included in our study were very likely to be skilled and committed goat caretakers. Despite that, they usually failed to notice clinical signs until SRLV infection had already been widespread in the herd. Therefore, only monitoring programs based on regular serological testing of a representative sample of goats in the herd would allow to detect the disease early enough to take effective control measures. Furthermore, only laboratory screening may constitute the trustworthy basis for official certification of goat herds as CAE-free e.g. in animal trading.

Obviously, arthritis is not the only clinical sign of SRLV infection. At least progressive weight loss is similarly common in infected goats [8] and has also been linked to SRLV infection occurrence in Poland [22]. However, we decided not to include progressive weight loss in our analysis as this clinical sign will rather make most farmers (and veterinarians) suspect gastrointestinal nematode infection or nutritional problems and will probably not be considered as indicating SRLV infection. Therefore, practical application of such analysis would be doubtful. On the other hand, we do not observe in Poland any other goat disease producing carpal arthritis but CAE, obviously except for sporadic mechanical injuries. Swollen carpal joints are therefore very specific hallmark of SRLV infection, which is most likely to concern farmers and encourage them to seek professional assistance. We limit ourselves in our study to carpal joints (called by Polish farmers “front knees”) as their swelling is most noticeable–indeed we have never met a farmer complaining about swelling of any other joints.

The answer “rarely” in our questionnaire means that the farmers have seen some goats (or maybe just a single one) with the swollen carpal joint, but it has not raised their concern. To suspect SRLV infection on the basis of a single arthritic goat in the herd a farmer, or a veterinarian asked for consultation, must be aware of the existence and importance of this disease. This factor is associated with the general quality of goat management and goat health care in the country, which in Poland is rather low. Goats are the least important farm animals for veterinarians in Poland, and this fact corresponds to farmers’ attitude towards veterinary service–famers rarely seek doctors’ advice assuming (not groundlessly) it barely could help. Therefore, from practical standpoint, we ought to assume that only the answer “often” signifies that in the farmer’s opinion there is a true health problem in the herd, which perhaps requires professional intervention.

There is a considerable amount of uncertainty regarding the true within-herd seroprevalence in our study demonstrated by quite wide confidence intervals (S1 Table). It stems both from determining apparent seroprevalence on the basis of a representative sample in a part of herds and from the imperfectness of the diagnostic tests used. If the true within-herd seroprevalence substantially differed the numerical results would change, however we did our utmost to keep the analysis free from any systematic bias so we doubt the final conclusion would change.

Concluding, our study clearly shows that when the farmer realizes that goats suffer from arthritis, SRLV infection is almost certainly already widespread in the herd. Therefore, even very careful observation of goats in the herd does not allow for early detection of SRLV infection.

## Supporting information

S1 Table Detailed data on 73 Polish goats herds included in the analysis. (DOCX)

S2 Table Interim stages of the forward stepwise procedure of developing the ordinal logistic regression model by entering additional explanatory variables: herd size and farmer’s experience in goat management. (DOCX)
