# Supplementary material for: Relationship between the dissemination of small ruminant lentivirus infection in goat herds and opinion of farmers on the occurrence of arthritis
Source: PLoS One. 2018 Sep 13;13(9):e0204134. doi: 10.1371/journal.pone.0204134 (PMC6136802; doi:10.1371/journal.pone.0204134)
Supplement: S1 Table — (DOCX) [file pone.0204134.s001.docx]

S1 Appendix. Detailed data on 73 Polish goats herds included in the analysis

| Herd | Herd size | Time since established = time for which the herd has been under the farmer's direct supervision | Collected sample size | Required sample size | ELISA used | Seropositive goats | Apparent within-herd seroprevalence | True within-herd seroprevalence  (CI 95%) | How often arthritis is observed in the herd? |
| --- | --- | --- | --- | --- | --- | --- | --- | --- | --- |
| 1 | 48 | 21 | 48 | 35 | ID screen | 3 | 6.3% | 5.7% (1.9%, 16.1%) | Never |
| 2 | 68 | 14 | 68 | 44 | ID screen | 1 | 1.5% | 0.4% (0%, 6.1%) | Never |
| 3 | 20 | 2 | 19 | 18 | ID screen | 3 | 15.8% | 16.2% (5.4%, 36.4%) | Never |
| 4 | 36 | 4 | 36 | 28 | ID screen | 3 | 8.3% | 8.0% (2.7%, 21.4%) | Never |
| 5 | 31 | 13 | 31 | 25 | ID screen | 11 | 35.5% | 38.0% (23.1%, 55.4%) | Never |
| 6 | 85 | 3 | 85 | 50 | ID screen | 3 | 3.5% | 2.7% (0.8%, 8.7%) | Never |
| 7 | 38 | 15 | 38 | 29 | ID screen | 1 | 2.6% | 1.7% (0.2%, 12.0%) | Never |
| 8 | 78 | 20 | 78 | 47 | ID screen | 1 | 1.3% | 0.2% (0%, 5.1%) | Never |
| 9 | 426 | 27 | 208 | 80 | ID screen | 198 | 95.2% | 100.0% (98.2%, 100%) | Often |
| 10 | 1150 | 24 | 364 | 107 | ID screen | 239 | 65.7% | 71.3% (66.4%, 75.7%) | Often |
| 11 | 49 | 29 | 49 | 35 | ID screen | 33 | 67.3% | 73.1% (59.4%, 83.5%) | Often |
| 12 | 38 | 35 | 31 | 28 | ID screen | 25 | 80.6% | 87.8% (72.0%, 95.3%) | Often |
| 13 | 138 | 10 | 135 | 64 | ID screen | 2 | 1.5% | 0.4% (0%, 3.5%) | Never |
| 14 | 78 | 10 | 75 | 47 | ID screen | 57 | 76.0% | 82.7% (72.6%, 89.6%) | Often |
| 15 | 108 | 2 | 105 | 57 | ID screen | 65 | 61.9% | 67.1% (57.7%, 75.4%) | Often |
| 16 | 61 | 12 | 60 | 41 | ID screen | 54 | 90.0% | 98.1% (90.8%, 99.6%) | Often |
| 17 | 80 | 3 | 77 | 48 | ID screen | 2 | 2.6% | 1.7% (0.3%, 7.6%) | Never |
| 18 | 77 | 3 | 74 | 47 | ID screen | 24 | 32.4% | 34.6% (24.8%, 45.9%) | Never |
| 19 | 62 | 10 | 59 | 41 | ID screen | 13 | 22.0% | 23.1% (14.2%, 35.3%) | Often |
| 20 | 101 | 18 | 100 | 55 | ID screen | 61 | 61.0% | 66.1% (56.4%, 74.6%) | Rarely |
| 21 | 88 | 15 | 85 | 51 | ID screen | 81 | 95.3% | 100.0% (95.7%, 100%) | Often |
| 22 | 120 | 8 | 116 | 60 | ID screen | 115 | 99.1% | 100.0% (96.8%, 100%) | Often |
| 23 | 82 | 9 | 80 | 49 | ID screen | 78 | 97.5% | 100.0% (95.4%, 100%) | Often |
| 24 | 80 | 4 | 76 | 48 | ID screen | 33 | 43.4% | 46.7% (35.9%, 57.8%) | Never |
| 25 | 104 | 15 | 102 | 56 | ID screen | 2 | 2.0% | 1.0% (0.2%, 5.3%) | Never |
| 26 | 139 | 21 | 135 | 64 | ID screen | 133 | 98.5% | 100.0% (97.2%, 100%) | Often |
| 27 | 85 | 12 | 82 | 50 | ID screen | 9 | 11.0% | 10.9% (5.8%, 19.5%) | Never |
| 28 | 98 | 22 | 97 | 54 | ID screen | 91 | 93.8% | 100.0% (96.2%, 100%) | Often |
| 29 | 171 | 1 | 171 | 70 | ID screen | 122 | 71.3% | 77.5% (70.7%, 83.1%) | Often |
| 30 | 102 | 1 | 102 | 55 | ID screen | 88 | 86.3% | 94.0% (87.6%, 97.2%) | Often |
| 31 | 127 | 13 | 127 | 61 | ID screen | 127 | 100.0% | 100.0% (97.1%, 100%) | Often |
| 32 | 101 | 20 | 101 | 55 | ID screen | 96 | 95.0% | 100.0% (96.3%, 100%) | Often |
| 33 | 126 | 16 | 125 | 61 | ID screen | 123 | 98.4% | 100.0% (97.0%, 100%) | Often |
| 34 | 40 | 17 | 40 | 30 | ID screen | 40 | 100.0% | 100.0% (91.2%, 100%) | Often |
| 35 | 39 | 9 | 39 | 30 | ID screen | 39 | 100.0% | 100.0% (91.0%, 100%) | Often |
| 36 | 62 | 1 | 62 | 41 | ID screen | 17 | 27.4% | 29.1% (19.2%, 41.3%) | Never |
| 37 | 55 | 3 | 55 | 38 | ID screen | 8 | 14.5% | 14.8% (7.8%, 26.5%) | Rarely |
| 38 | 44 | 2 | 44 | 32 | ID screen | 6 | 13.6% | 13.8% (6.5%, 26.9%) | Never |
| 39 | 94 | 12 | 94 | 53 | ID screen | 24 | 25.5% | 27.0% (19.0%, 36.7%) | Never |
| 40 | 20 | 9 | 20 | 18 | ID screen | 1 | 5.0% | 4.3% (0.7%, 22.6%) | Never |
| 41 | 42 | 11 | 42 | 31 | ID screen | 35 | 83.3% | 90.8% (78.3%, 96.4%) | Never |
| 42 | 47 | 2 | 47 | 34 | ID screen | 11 | 23.4% | 24.6% (14.5%, 38.5%) | Never |
| 43 | 22 | 10 | 22 | 19 | ID screen | 15 | 68.2% | 74.0% (53.2%, 87.7%) | Never |
| 44 | 73 | 27 | 73 | 45 | ID screen | 15 | 20.5% | 21.5% (13.6%, 32.2%) | Never |
| 45 | 51 | 1 | 49 | 36 | ID screen | 2 | 4.1% | 3.3% (0.8%, 12.6%) | Never |
| 46 | 134 | 23 | 134 | 63 | ID screen | 112 | 83.6% | 91.0% (85.0%, 94.8%) | Often |
| 47 | 184 | 1 | 184 | 72 | ID screen | 26 | 14.1% | 14.4% (10.0%, 20.2%) | Never |
| 48 | 80 | 3 | 80 | 48 | ID screen | 54 | 67.5% | 73.3% (62.7%, 81.8%) | Rarely |
| 49 | 36 | 14 | 35 | 27 | Checkit | 4 | 11.4% | 11.0% (4.3%, 25.4%) | Rarely |
| 50 | 124 | 5 | 120 | 56 | Checkit | 25 | 20.8% | 20.6% (14.3%, 28.7%) | Often |
| 51 | 62 | 3 | 62 | 39 | Checkit | 3 | 4.8% | 4.2% (1.4%, 12.4%) | Never |
| 52 | 50 | 4 | 50 | 34 | Checkit | 19 | 38.0% | 38.1% (25.9%, 51.9%) | Never |
| 53 | 20 | 6 | 20 | 17 | Checkit | 2 | 10.0% | 9.5% (2.6%, 29.5%) | Never |
| 54 | 54 | 7 | 54 | 36 | Checkit | 43 | 79.6% | 80.6% (68.2, 89.0%) | Rarely |
| 55 | 35 | 4 | 35 | 26 | Checkit | 1 | 2.9% | 2.2% (0.3%, 13.5%) | Never |
| 56 | 244 | 7 | 244 | 72 | Checkit | 187 | 76.6% | 77.6% (71.9%, 82.4%) | Rarely |
| 57 | 36 | 4 | 36 | 27 | Checkit | 20 | 55.6% | 56.0% (40.0%, 70.9%) | Rarely |
| 58 | 31 | 9 | 31 | 24 | Checkit | 10 | 32.3% | 32.2% (18.6%, 49.8%) | Never |
| 59 | 38 | 3 | 38 | 28 | Checkit | 25 | 65.8% | 66.5% (50.6%, 79.4%) | Rarely |
| 60 | 146 | 4 | 146 | 60 | Checkit | 41 | 28.1% | 28.0% (21.3%, 35.7%) | Often |
| 61 | 22 | 4 | 22 | 19 | Checkit | 8 | 36.4% | 36.4% (19.8%, 57.1%) | Rarely |
| 62 | 68 | 7 | 68 | 41 | Checkit | 11 | 16.2% | 15.8% (9.0%, 26.3%) | Never |
| 63 | 71 | 6 | 71 | 42 | Checkit | 6 | 8.5% | 7.9% (3.6%, 16.6%) | Never |
| 64 | 33 | 8 | 33 | 25 | Checkit | 1 | 3.0% | 2.4% (0.4%, 14.3%) | Never |
| 65 | 28 | 11 | 28 | 22 | Checkit | 1 | 3.6% | 2.9% (0.5%, 16.8%) | Never |
| 66 | 22 | 7 | 22 | 19 | Checkit | 13 | 59.1% | 59.6% (39.2%, 77.2%) | Never |
| 67 | 24 | 10 | 24 | 20 | Checkit | 1 | 4.2% | 3.5% (0.6%, 19.3%) | Never |
| 68 | 62 | 5 | 34 | 39 | Checkit | 3 | 8.8% | 8.3% (2.8%, 22.3%) | Never |
| 69 | 41 | 4 | 41 | 30 | Checkit | 10 | 24.4% | 24.2% (13.7%, 39.1%) | Never |
| 70 | 93 | 9 | 93 | 49 | Checkit | 91 | 97.8% | 99.2% (94.7%, 99.9%) | Never |
| 71 | 58 | 7 | 58 | 37 | Checkit | 29 | 50.0% | 50.4% (37.9%, 62.8%) | Often |
| 72 | 22 | 9 | 21 | 19 | Checkit | 13 | 61.9% | 62.5% (41.4%, 79.7%) | Never |
| 73 | 38 | 6 | 30 | 28 | Checkit | 1 | 3.3% | 2.7% (0.4%, 15.7%) | Never |
