# Supplementary material for: Relationship between the dissemination of small ruminant lentivirus infection in goat herds and opinion of farmers on the occurrence of arthritis
Source: PLoS One. 2018 Sep 13;13(9):e0204134. doi: 10.1371/journal.pone.0204134 (PMC6136802; doi:10.1371/journal.pone.0204134)
Supplement: S2 Table — (DOCX) [file pone.0204134.s002.docx]

S2 Appendix. Interim stages of the forward stepwise procedure of developing the ordinal logistic regression model by entering additional explanatory variables: herd size and farmer’s experience in goat management

| Variable | Regression coefficient (b) | Standard error (SE) | 95% confidence interval | Wald χ^2^ statistics | p-value |
| --- | --- | --- | --- | --- | --- |
| Model with herd size | | | | | |
| Arthritis |  |  |  |  |  |
| never observed (0) | - | - | - | - | - |
| rarely observed (1) | 3.353 | 0.749 | 1.885, 4.821 | 20.04 | <0.001 |
| often observed (2) | 4.490 | 0.865 | 2.794, 6.186 | 26.93 | <0.001 |
| True within-herd seroprevalence (TP) | 0.052 | 0.010 | 0.033, 0.072 | 27.53 | <0.001 |
| Herd size (HS) | 0.010 | 0.007 | -0.003, 0.024 | 2.18 | 0.140 |
| Model with farmer’s experience in goat management | | | | | |
| Arthritis |  |  |  |  |  |
| never observed (0) | - | - | - | - | - |
| rarely observed (1) | 3.074 | 0.657 | 1.786, 4.361 | 21.89 | <0.001 |
| often observed (2) | 4.155 | 0.772 | 2.642, 5.667 | 28.99 | <0.001 |
| True within-herd seroprevalence (TP) | 0.053 | 0.010 | 0.034, 0.073 | 27.91 | <0.001 |
| Time since established | 0.045 | 0.041 | -0.036, 0.126 | 1.18 | 0.278 |
